# Facile Synthesis of Polyaniline/Carbon-Coated Hollow Indium Oxide Nanofiber Composite with Highly Sensitive Ammonia Gas Sensor at the Room Temperature

**DOI:** 10.3390/s22041570

**Published:** 2022-02-17

**Authors:** Sheng-Zhe Hong, Qing-Yi Huang, Tzong-Ming Wu

**Affiliations:** Department of Materials Science and Engineering, National Chung Hsing University, 250 Kuo Kuang Road, Taichung 402, Taiwan; gsn30628@gmail.com (S.-Z.H.); jordan20310687@gmail.com (Q.-Y.H.)

**Keywords:** polyaniline, hollow carbon-coated indium trioxide nanofiber, ammonia gas sensor, composite

## Abstract

Hollow carbon-coated In_2_O_3_ (C#In_2_O_3_) nanofibers were prepared using an efficiently combined approach of electrospinning, high-temperature calcination, and hydrothermal process. The polyaniline (PANI)/hollow C#In_2_O_3_ nanofiber composites were synthesized used hollow C#In_2_O_3_ nanofibers worked as a core through the in situ chemical oxidative polymerization. The morphology and crystalline structure of the PANI/hollow C#In_2_O_3_ nanofiber composite were identified using wide-angle X-ray diffraction and transmission electron microscopy. The gas-sensing performances of the fabricated PANI/hollow C#In_2_O_3_ nanofiber composite sensor were estimated at room temperature, and the response value of the composite sensor with an exposure of 1 ppm NH_3_ was 18.2, which was about 5.74 times larger than that of the pure PANI sensor. The PANI/hollow C#In_2_O_3_ nanofiber composite sensor was demonstrated to be highly sensitive to the detection of NH_3_ in the concentration range of 0.6~2.0 ppm, which is critical for kidney or hepatic disease detection from the human breath. This composite sensor also displayed superior repeatability and selectivity at room temperature with exposures of 1.0 and 2.0 ppm NH_3_. Because of the outstanding repeatability and selectivity to the detection of NH_3_ at 1.0 and 2.0 ppm confirmed in this investigation, the PANI/hollow C#In_2_O_3_ nanofiber composite sensor will be considered as a favorable gas-sensing material for kidney or hepatic disease detection from human breath.

## 1. Introduction

Human breath contains a mixture of nitrogen, oxygen, carbon dioxide, water, and other gas compounds occurring in concentrations ranging from a few ppt to thousands of ppm [1,2]. An adjustment in component is strongly reliant on several topics, for instance age, health condition, and gender. Among these gas compounds, ammonia (NH_3_) is a breakdown product of protein, which is normally transferred into urea by the liver and exhausted through the kidneys. Therefore, the change of NH_3_ composition in human breath might show specific relationships regarding kidney or hepatic disease [3,4,5,6,7]. According to a previous study, the concentrations of NH_3_ of healthy human breath are numerous hundred ppb and significantly increase to several ppm during either kidney or hepatic collapses. During this work, Turner et al. [8] reported that a NH_3_ concentration larger than 1.6 ppm is identified be unhealthy, while less than 1.1 ppm is considered as healthy. An intermediate concentration between the borderline concentration of unhealthy and healthy is regarded for charity. Consequently, NH_3_ gas sensors within the special limit are receiving a lot of attention.

Intrinsically conducting polymers (ICPs) containing the excellent electronic transfer between ICPs and gas molecules with increasing gas vapor adsorption have been applied as a key component for sensing applications [9]. Among ICPs, polyaniline (PANI) is widely used for sensing areas owing to its excellent responsivity to NH_3_, high conductivity, easy synthesis, remarkable doping/de-doping chemical reaction, and outstanding environmental stability [10]. According to previous investigations, the combination of metal oxide n-type semiconductors, such as CeO_2_, In_2_O_3_, SnO_2_, and WO_3_, or carbon-based materials into PANI can enhance the stability, sensitivity, and repeatability of the sensor nanocomposite sensor [11,12,13,14]. Xue et al. [12] reported a PANI/carbon nanotube (CNT) composite showed enhanced sensing performance and stability at room temperature as compared to that of pure PANI. Li et al. [14] prepared a composite sensor with improved sensing performance at room temperature using PANI and flower-like WO_3_ with higher special surface area. Recently, Wu et al. [15] used In_2_O_3_ nanoparticles, graphene nanoribbon (GNR), and PANI to synthesize a composite sensor containing nanostructured conformation. These results revealed that the sensing properties at room temperature were considerably greater than those of pure PANI and PANI/GNR composite sensor. Recently, Wu et al. [16] applied a high special surface area hollow In_2_O_3_ nanofiber, nitrogen-doped graphene quantum dot (N-GQD), and PANI to synthesize a ternary composite. Their results revealed that the sensing properties of composite sensor at room temperature were greater than those of PANI/hollow In_2_O_3_ nanofiber composites. The superior gas-sensing performances were attributed to the formation of p–n junction between the p-type PANI and n-type high special surface area hollow In_2_O_3_ nanofiber with increasing electron depletion layer, as well as the sensing response.

This work describes a simple fabrication of PANI/hollow carbon-coated In_2_O_3_ (C#In_2_O_3_) nanofiber as an electrode employed as gas-sensing material to detect ammonia in the concentration range of 0.6~2.0 ppm from the human breath. To our knowledge, no report on PANI and carbon-coated surface of In_2_O_3_ nanofiber composite with one-step process and larger special surface area has been published. Consequently, the synthesized material is anticipated to show bettered gas-sensing properties and exceptional repeatability and selectivity. The structure, morphology, and gas-sensing performances of the manufactured composites are considerably characterized in the following discussion.

## 2. Experimental

### 2.1. Materials

Polyvinylpyrrolidone (PVP), isopropyl alcohol (>98%), and ammonium persulfate (APS, >98%) were obtained from JT-Baker Chemical Company (Phillipsburg, NJ, USA). Indium(III) nitrate hydrate (In(NO_3_)_3_, >99.9%), citric acid (CA, >98%), sulfuric acid (>98%), urea (>98%), and aniline monomer were purchased from Sigma-Aldrich Chemical Company (St. Louis, MO, USA). All chemicals were utilized as received.

### 2.2. Synthesis of Polyaniline/Carbon-Coated Hollow Indium Trioxide Nanofiber Composites

The hollow In_2_O_3_ nanofibers were prepared using indium nitrate hydrate as indium source. In a usual preparation process, 1.1 g In(NO_3_)_3_ and 3.5 g PVP were mixed in 10.6 mL DMF and 12 mL ethyl alcohol, and the solution was stirred to completely dissolve In(NO_3_)_3_ and PVP for 10 h. Then, the mixed solution was loaded into a 20 mL syringe containing a metallic needle with 0.5 mm diameter for electrospinning process [17,18]. The tip of the metallic needle was applied to 20 kV high-voltage power with a feeding rate of 0.3 mL/h, and the distance between the collector and the needle was approximately 15 cm. Following the electrospinning process for 24 h, the fabricated as-spun nanofiber was thermally calcinated for 3 h at 800 °C with a heating rate of 5 °C/min to prepare hollow In_2_O_3_ nanofibers.

For the hydrothermal synthesis of the core–shell structure of carbon-coated hollow indium trioxide (C#In_2_O_3_) nanofiber, 0.18 g urea, 0.21 g CA, and 0.05 g hollow In_2_O_3_ nanofiber were mixed under stirring in a 10 mL beaker for 30 min at room temperature. Then the mixed solution was loaded into a poly(tetrafluoroethylene) reactor and heated for 4 h at 160 °C. The obtained products were further modified by adding ethanol into the solution and centrifuging for 2 h at 5000 rpm to attain the C#In_2_O_3_ samples. The fabricated product was washed by distilled water (DI water) and consequently purified using dialysis bag for 24 h.

In situ chemical oxidative polymerization was used to synthesize the polyaniline (PANI)/hollow C#In_2_O_3_ nanofiber composites. The in situ chemical oxidative polymerization mechanism of PANI can be divided into two steps. First, the aniline monomer is oxidized to form cationic radicals followed by free radical polymerization. The achieved aniline dimer consequently experiences a deprotonation process, resulting in an active neutral dimer, which facilitates the dimer to react in the following oxidation process. This process is repeated, leading eventually to the formation of PANI. In a typical preparation process, a certain weight ratio of hollow C#In_2_O_3_ nanofiber was dispersed in 50 mL HCl solution and sonicated for 2 h. Consequently, the aniline monomer was added into the dispersed solution of the hollow C#In_2_O_3_ nanofiber and stirred for 1 h. The APS was then dissolved in 20 mL HCl solution and was slowly added into the mixed aniline monomer/hollow C#In_2_O_3_ nanofiber solution. The reactants were polymerized at 0 °C for 3 h. The obtained product was filtered, washed several times with DI water and methanol, and vacuum dried at 60 °C for 24 h. The yield of sample preparation is about 86%.

### 2.3. Material Characterization

The structure of the synthesized PANI/hollow C#In_2_O_3_ nanofiber composites was measured by wide-angle X-ray diffraction (WAXD) and Fourier transform infrared (FTIR). WAXD measurements operated using X-ray diffractometer (Bruker D8, BRUKER AXS, Inc., Madison, WI, USA) with a Ni-filtered Cu Kα radiation were recorded at 2θ ranging from 1.5° to 40° with an increment of 1°/min. FTIR spectra in a range of 400–4000 cm^−1^ were determined using a Perkin-Elmer Spectrum One spectrometer (Waltham, MA, USA). The morphology of all specimens was characterized by transmission electron microscopy (TEM) and field-emission scanning electron microscopy (FESEM). The TEM experiment was measured by JEOL JEM-2010 (JEOL Ltd., Tokyo, Japan). Specimens of TEM experiments were made by a Reichert Ultracut ultramicrotome. The FESEM measurement was performed by a JEOL JSM-6700F field-emission instrument (JEOL Ltd., Tokyo, Japan). Gold was used to coat the surface of all samples to avoid charging. Raman spectra were recorded under a Renishaw system 1000 using an Argon ion laser operating at 514.5 nm with a CCD detector. The BET and BJH methods using gas sorption analyzer (Quantachrome AutoSorb IQ, Montgomeryville, PA, USA) were used to determine the specific surface area obtained using N_2_ sorption isotherms.

### 2.4. Gas-Sensing Properties

The gas-sensing performance of the sensors was determined at 25 °C using a homemade dynamic test system with a simultaneous resistance acquisition stage. The gas concentrations of interfering gas samples including CH_3_OH, C_2_H_5_OH, C_3_H_6_O, and C_6_H_14_ and targeting NH_3_ sample were determined by changing the test samples and nitrogen mixing ratio. The equation of R = R_g_/R_a_ is used to calculate the sensor response, where R_g_ and R_a_ are the sensor resistances in test gases and air, respectively. The sensitivity is obtained from the slope of the response–concentration fitting curve.

## 3. Results

### 3.1. Structural and Morphological Characterizations

**Figure 1:**
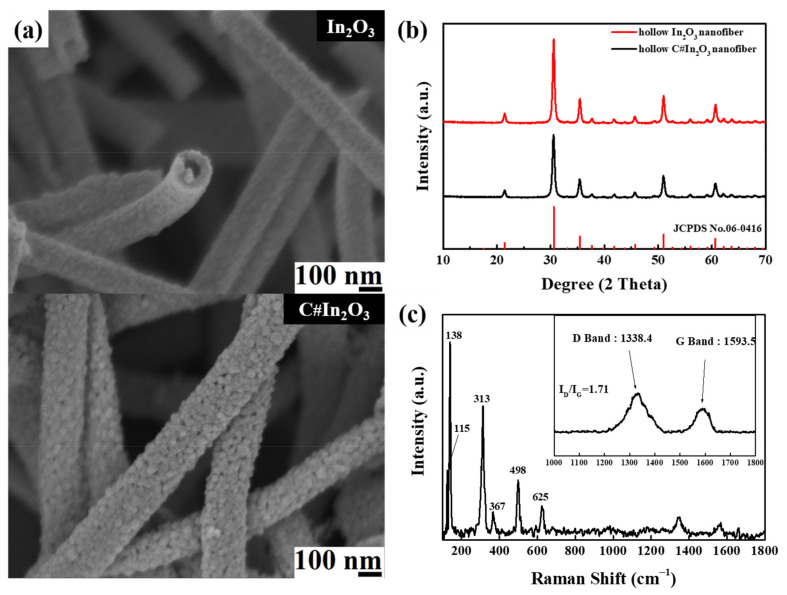
(**a**) SEM images of hollow In_2_O_3_ and C#In_2_O_3_ nanofibers, (**b**) WAXD data of hollow In_2_O_3_ and C#In_2_O_3_ nanofibers, and (**c**) Raman spectra of hollow and C#In_2_O_3_ nanofibers.

The characteristic SEM micrographs of hollow In_2_O_3_ and C#In_2_O_3_ nanofibers are illustrated in Figure 1a. The hollow In_2_O_3_ nanofiber displays a continuous hollow and fibrous morphology with coarse surface. After carbon-coating the surface of the hollow In_2_O_3_ nanofiber, the particle-like morphology is observed, and the surface becomes rougher. The average diameter of the hollow In_2_O_3_ nanofiber was about 165 nm and was slightly increased to 190 nm for the hollow C#In_2_O_3_ nanofiber. The WAXD technique was used to characterize the crystalline structure of the hollow In_2_O_3_ and C#In_2_O_3_ nanofibers. Both WAXD diffraction profiles of hollow In_2_O_3_ and C#In_2_O_3_ nanofibers, as exhibited in Figure 1b, present five intense diffraction peaks at 2θ = 21.7°, 30.6°, 35.4°, 51.2°, and 60.7°, designated to (211), (222), (400), (440), and (622) planes of crystalline In_2_O_3_, respectively. This result recommends that the crystalline structure of hollow In_2_O_3_ nanofibers is determined to be a cubic crystal phase [15], and the carbon-coated process did not change the crystalline structure of the In_2_O_3_ nanofiber. The absorption bands of hollow C#In_2_O_3_ nanofiber obtained by using the Raman spectra were presented in Figure 1c. Two intense absorption peaks at 1587 cm^−1^ (G mode) and at 1345 cm^−1^ (D mode) are obtained in this figure. The I_D_/I_G_ ratio is 1.71, which indicates that an amorphous structure of carbon-coated material was obtained.

**Figure 2:**
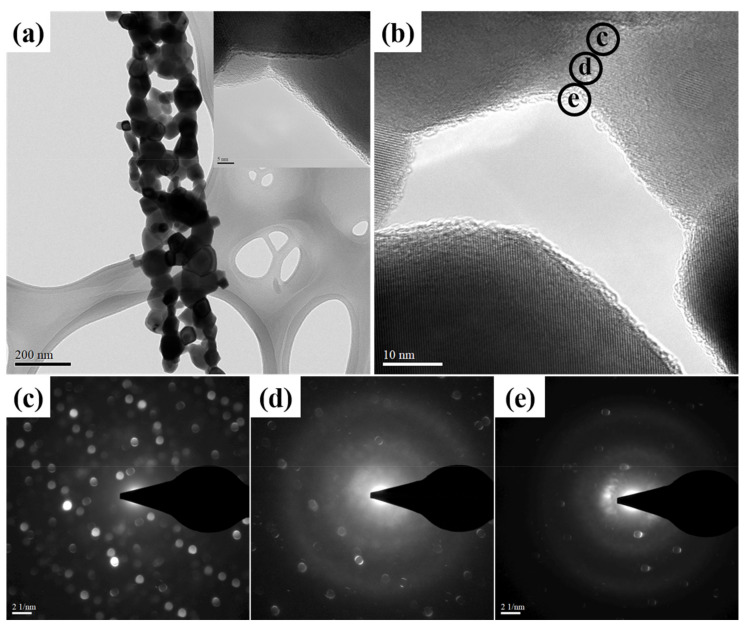
(**a**) TEM image of hollow C#In_2_O_3_ nanofibers. (**b**) High-magnification TEM image of hollow C#In_2_O_3_ nanofibers. Nano beam diffraction (NBD) images of selected area (**c**–**e**) in Figure 2b.

**Figure 3:**
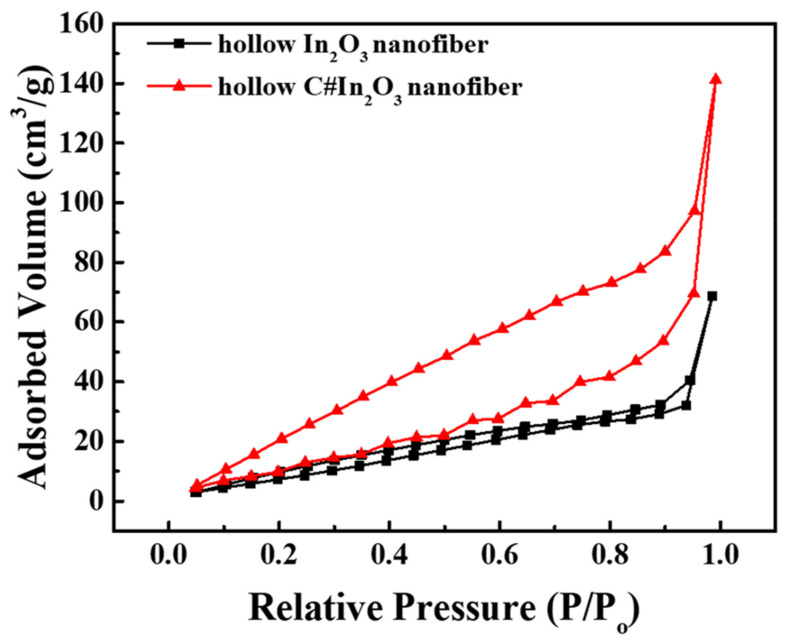
The BET curves of hollow In_2_O_3_ and C#In_2_O_3_ nanofibers.

In order to identify the carbon-coated morphology of C#In_2_O_3_ nanofiber, the high-resolution TEM with nano beam diffraction (NBD) mode shown in Figure 2 was applied to obtain these evidence. From Figure 2a,b, it is clear that there is a thin layer with lower electron density coated on the surface of higher electron density material, which is identified as the carbon-coated material and In_2_O_3_ nanofiber, respectively. In addition, the microstructure of carbon-coated material and In_2_O_3_ nanofiber were further identified by NBD, as illustrated in Figure 2c–e. From the interface of the hollow In_2_O_3_ nanofiber, as shown in Figure 2c, there are a lot of diffraction spots. These results indicate that the microstructure of hollow In_2_O_3_ nanofibers is crystalline, which is consistent with the WAXD data. As the nano beam diffraction focuses to the lower electron density layer, as shown in Figure 2e, few diffraction spots are observed. This result indicates that the microstructure of carbon-coated material is amorphous, which is consistent with the Raman data. Figure 3 reveals the specific surface area of the hollow In_2_O_3_ and C#In_2_O_3_ nanofibers. The data of the specific surface area for the hollow In_2_O_3_ and C#In_2_O_3_ nanofibers are 39.6 and 55.2 m^2^ g^−1^, respectively. The specific surface area significantly increases with the carbon-coated materials on the surface of the hollow In_2_O_3_ nanofiber. This observation suggests that the carbon-coated hollow In_2_O_3_ nanofibers would provide more reaction site for further interaction.

**Figure 4:**
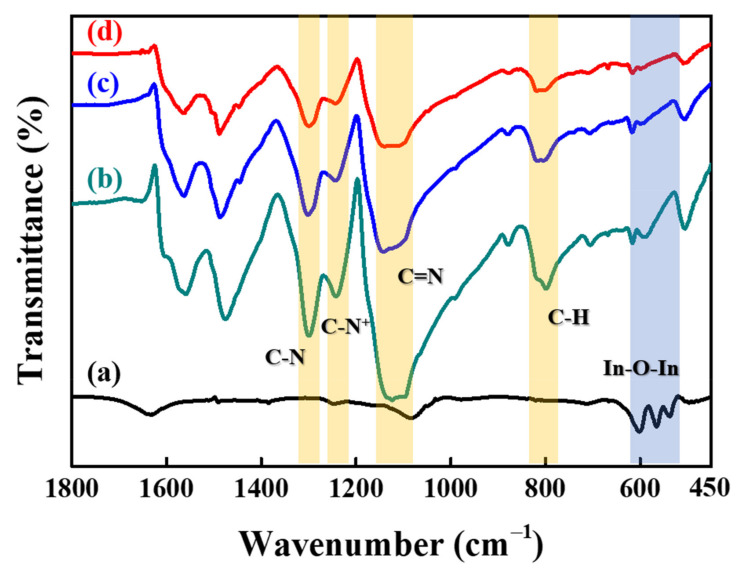
FT-IR spectra of (**a**) hollow In_2_O_3_ nanofiber, (**b**) pure PANI matrix, (**c**) PANI/hollow In_2_O_3_ nanofiber, and (**d**) PANI/hollow C#In_2_O_3_ nanofiber composites.

The FT-IR and TEM methods were used to characterize the chemical structure and morphology of the synthesized polyaniline (PANI) coated on the surface of hollow In_2_O_3_ and C#In_2_O_3_ nanofibers. Figure 4 reveals the FT-IR spectra of PANI coated on the surface of hollow In_2_O_3_ and C#In_2_O_3_ nanofiber composites. The FT-IR data of pure PANI and hollow In_2_O_3_ nanofiber are also displayed in this figure. The characteristic peaks of hollow In_2_O_3_ nanofiber observed at 538, 567, and 600 cm^−1^ contributed to the In–O–In stretching vibration. The absorption peak of PANI occurring at 1240 cm^−1^ was ascribed to the C–N^●+^ stretching vibration, and the characteristic peak at 800 cm^−1^ was attributed to a C–H out-of-plane bending vibration of the 1,4-disubstituted aromatic rings [19]. The absorption peaks of C=N and C–N stretching vibrations were obtained at 1112 and 1294 cm^−1^, respectively. The FT-IR spectra of PANI-coated hollow In_2_O_3_ and C#In_2_O_3_ nanofibers were almost indistinguishable to those of neat PANI, suggesting that the surface of the hollow In_2_O_3_ and C#In_2_O_3_ nanofibers was coated with PANI to form PANI/hollow In_2_O_3_ nanofiber and PANI/hollow C#In_2_O_3_ nanofiber composites.

**Figure 5:**
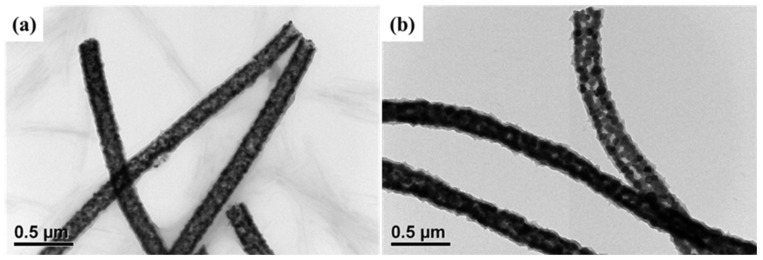
TEM images of (**a**) PANI/hollow In_2_O_3_ nanofiber and (**b**) PANI/hollow C#In_2_O_3_ nanofiber composites.

**Figure 6:**
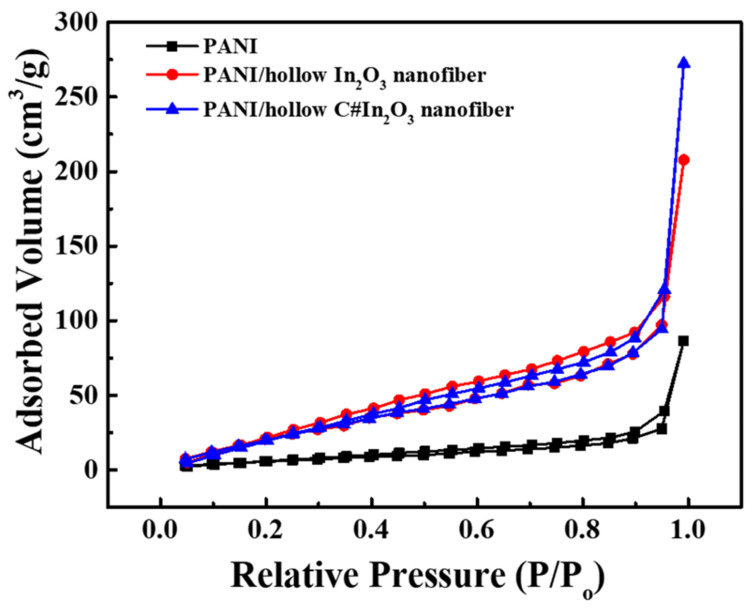
The BET profiles of PANI, PANI/hollow In_2_O_3_ nanofiber, and PANI/hollow C#In_2_O_3_ nanofiber.

Figure 5 shows the TEM images of PANI/hollow In_2_O_3_ nanofiber and PANI/hollow C#In_2_O_3_ nanofiber composites. By coating conductive PANI on the surface of the hollow In_2_O_3_ and C#In_2_O_3_ nanofiber, the diameters of the PANI/hollow In_2_O_3_ nanofiber and PANI/hollow C#In_2_O_3_ nanofiber composites were slightly increased, compared to the hollow In_2_O_3_ and C#In_2_O_3_ nanofiber. The diameters of the fabricated composites increased from 165 nm and 190 nm for hollow In_2_O_3_ and C#In_2_O_3_ nanofiber to 190 and 220 nm for PANI/hollow In_2_O_3_ nanofiber and PANI/hollow C#In_2_O_3_ nanofiber composites, respectively. The increasing diameter in the fabricated composites can be attributed to a thin coating layer of PANI on the surface of the hollow In_2_O_3_ and C#In_2_O_3_ nanofiber. Figure 6 reveals the specific surface area of the PANI/hollow In_2_O_3_ nanofiber and PANI/hollow C#In_2_O_3_ nanofiber composites. The data of the specific surface area for the PANI/hollow In_2_O_3_ nanofiber and PANI/hollow C#In_2_O_3_ nanofiber composites are 24.8, 102.1, and 111.6 m^2^ g^−1^. The specific surface area of both composite sensors is significantly higher than that of pure polymer matrix sensor. The specific surface area of PANI/hollow C#In_2_O_3_ nanofiber composite is relatively higher than that of PANI/hollow In_2_O_3_ nanofiber composite. This observation suggests that the PANI/hollow C#In_2_O_3_ nanofiber composite would provide more reaction site for further interaction, compared to that of the PANI/hollow In_2_O_3_ nanofiber composite.

### 3.2. NH_3_-Sensing Performance

**Figure 7:**
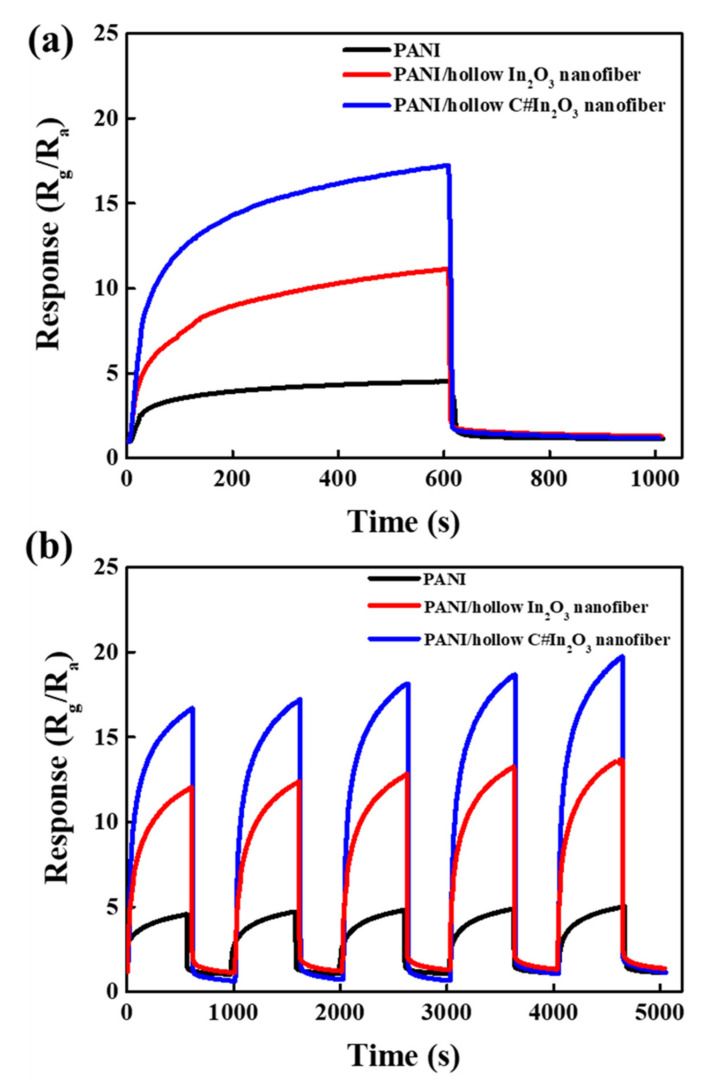
(**a**) The room-temperature response and (**b**) the repeatability and reversibility of PANI, PANI/hollow In_2_O_3_ nanofiber, and PANI/hollow C#In_2_O_3_ nanofiber composite sensors with exposure of 1 ppm NH_3_.

In order to evaluate the effect of carbon-coated material on the surface of hollow In_2_O_3_ nanofiber on the NH_3_-sensing property of the composite sensor, the response and recovery of PANI, PANI/hollow In_2_O_3_ nanofiber, and PANI/hollow C#In_2_O_3_ nanofiber sensors were completely examined. Figure 7 shows the dynamic response–recovery profiles of the PANI and composite sensors with exposure to 1 ppm NH_3_ at room temperature. These results indicated that all sensors reacted with a significant improvement in resistance when exposed to NH_3_, and the resistance fell down to the initial situation after the NH_3_ was switched to dry air. This outcome displays a representative performance and an excellent reversibility of the composite sensors. Exceptionally, the PANI/hollow# In_2_O_3_ nanofiber sensor demonstrated extremely greater response values than the pure PANI and PANI/hollow In_2_O_3_ nanofiber sensor, suggesting that hollow C#In_2_O_3_ nanofiber performs a dominant role in NH_3_-sensing measurements. The response values of pure PANI, PANI/hollow In_2_O_3_ nanofiber, and PANI/hollow C#In_2_O_3_ nanofiber sensors were about 3.6, 11.2, and 18.2, respectively. The response value of the previous investigation using PANI/N-GQD/hollow In_2_O_3_ nanofiber was 15.6 [16]. It is clear that the surface coated by a thin carbon layer showed a better gas sensing property. The enhancement of the sensing properties was assigned to the presence of a p–n heterojunction generated between the p-type PANI and n-type hollow C#In_2_O_3_ nanofiber [15,19]. The sensing repeatability and reversibility of the PANI, PANI/hollow In_2_O_3_ nanofiber, and PANI/hollow C#In_2_O_3_ nanofiber sensors to 1.0 ppm NH_3_ are presented in Figure 7b. The response values of all gas sensors dropped down to the initial response value after exposure to 1.0 ppm NH_3_. In the process of five continuous cycles, this typical behavior of response and recovery to 1.0 ppm NH_3_ approved exceptional reproducibility. This result suggests that the good repeatability of the PANI/hollow C#In_2_O_3_ nanofiber sensor is achieved.

**Figure 8:**
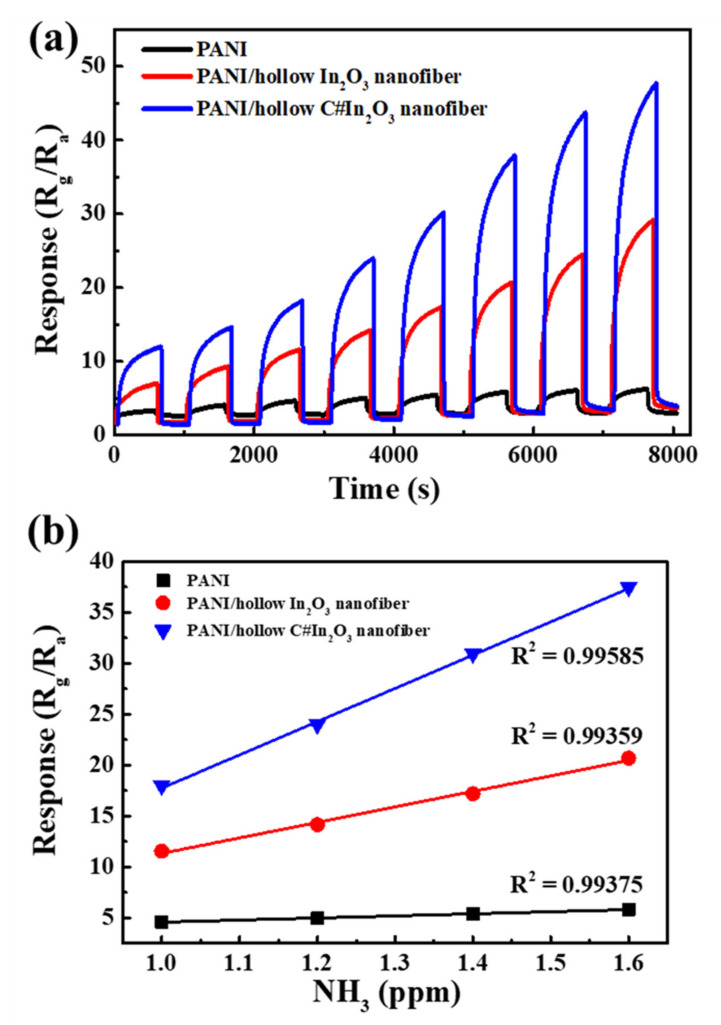
The (**a**) dynamic response–recovery curves shown in the concentration between 0.6 ppm and 2.0 ppm, and (**b**) the fitting curves of response versus concentration for PANI, PANI/hollow In_2_O_3_ nanofiber, and PANI/hollow C#In_2_O_3_ nanofiber composites.

In order to investigate the fabricated sensor used in the detection of human breath for kidney or hepatic disease, all sensors were operated to detect the NH_3_ at room temperature in the concentration between 0.6 ppm and 2.0 ppm. Figure 8a shows the NH_3_-sensing performance of the PANI, PANI/hollow In_2_O_3_ nanofiber, and PANI/hollow C#In_2_O_3_ nanofiber sensors. All results reveal that the response of each sensor instantaneously rose with increasing the exposure to NH_3_ and then, extremely, came back to the initial response value after exposure to dry air. The response of each sensor was extensively enhanced as the concentration of the analyst increased. These results represent that the response tendency of three sensors was approximately identical, but the values of response for all sensors were extremely different at the same concentration. It is clear that the PANI/hollow C#In_2_O_3_ nanofiber sensor possessed the highest response among the three sensors. The response values of the PANI/hollow C#In_2_O_3_ nanofiber sensors were correspondingly around 11.5, 14.2, 17.8, 23.5, 29.7, 37.5, 43.3, and 47.3 regarding to the concentration of 0.6, 0.8, 1.0, 1.2, 1.4, 1.6, 1.8, and 2.0 ppm. The value of response for the PANI/hollow C#In_2_O_3_ nanofiber sensor exposed at 1.0 ppm NH_3_ was correspondingly about 5.74 and 1.6 times greater than those of PANI and PANI/hollow In_2_O_3_ nanofiber sensor. The fitting curves of response versus concentrations of NH_3_ for three sensors are shown in Figure 8b. According to these profiles, the correlations between the values of response and the concentrations of NH_3_ are approximately linear. The matching functions were correspondingly dedicated as y = 2.04x + 2.55, y = 15.23x − 3.88, and y = 32.68x − 14.93 for the PANI, PANI/hollow In_2_O_3_ nanofiber, and PANI/hollow C#In_2_O_3_ nanofiber sensors. The correlation coefficients, R^2^, were also shown in this figure. The slopes of these related lines, classified as the sensitivity of the sensors, indicate that the sensitivity of the PANI/hollow C#In_2_O_3_ nanofiber sensors were larger than those of PANI and PANI/hollow In_2_O_3_ nanofiber sensors. These results suggest that the ability to detect NH_3_ using the PANI/hollow C#In_2_O_3_ nanofiber sensor is excellent, and this composite sensor is suitable for use as a favorable material for NH_3_ gas detection. Table 1 shows a comparison of the sensing properties of PANI/hollow C#In_2_O_3_ nanofiber and formerly reported sensors from the literature. Liu et al. [20] used MoS_2_ nanosheets, SnO_2_ nanotubes, and PANI to fabricate a composite sensor containing nanostructured conformation. Their result revealed that the response value of 50 ppm NH_3_ at room temperature was 7.5, which was lower than that of our results. Li et al. [14] prepared a composite sensor using PANI and flower-like WO_3_ with higher special surface area. Their data indicated that the response value of 10 ppm NH_3_ at room temperature was 7.14, which was also lower than that of our results. Therefore, it is clear that the PANI/hollow C#In_2_O_3_ nanofiber sensor investigated in this study showed superior sensing property to NH_3_ at room temperature than formerly reported sensors. Subsequently, a combination of p-type PANI and n-type hollow C#In_2_O_3_ nanofibers is proven as a powerful methodology for enhancing the NH_3_-sensing response of sensors.

**Figure 9:**
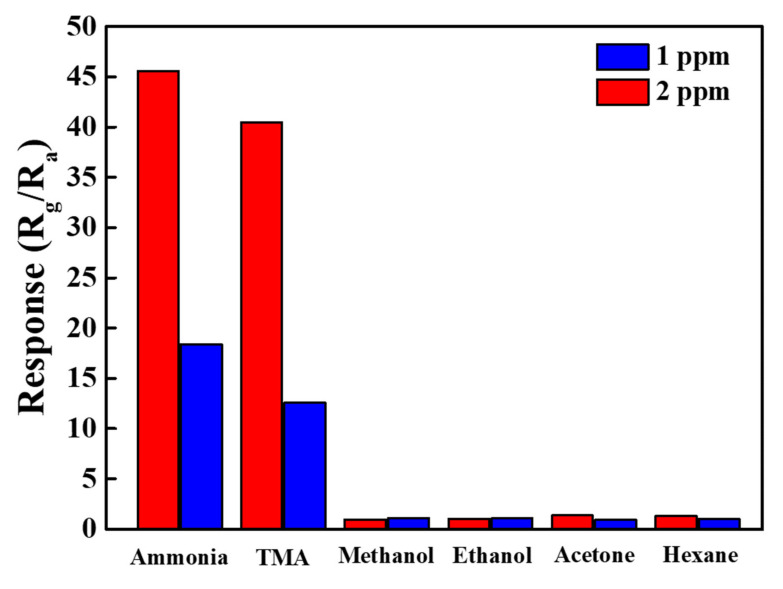
The selectivity of the PANI/hollow C#In_2_O_3_ nanofiber sensor toward NH_3_, trimethylamine (TMA), methanol, ethanol, acetone, and hexane with a concentration of 1 and 2 ppm.

The reversibility, repeatability, and selectivity of fabricated gas sensors are crucial for practicable applications. In reality, the gas sensors are generally exposed to plentiful analysts, and the target analyst is supposedly detected correctly without being affected by other analysts. The selectivity of the PANI/hollow C#In_2_O_3_ nanofiber sensor for ammonia, methanol, ethanol, acetone, and hexane with an exposure to the concentration of 1.0 and 2.0 ppm is shown in Figure 9. From this result, it was clear that the PANI/hollow C#In_2_O_3_ nanofiber sensor contained a high-level response performance to ammonia and trimethylamine (TMA) but revealed almost no response versus other gases. The surface absorption of amine group of NH_3_ and TMA on the interface of the PANI/hollow C#In_2_O_3_ nanofiber sensor may contribute to major mechanism of NH_3_ and TMA selectivity. The de-doping response between NH_3_/TMA and PANI plays a significant role in enhancing NH_3_- and TMA-sensing properties, indicating a selective response to NH_3_ [15,27]. Consequently, the PANI/hollow C#In_2_O_3_ nanofiber sensor displayed excellent selectivity concerning NH_3_ and TMA, versus other gases at room temperature.

## 4. Conclusions

Excellent gas-sensing properties of PANI/hollow C#In_2_O_3_ nanofiber composites were successfully prepared using in situ chemical oxidation polymerization. The gas-sensing performances of the fabricated PANI/hollow C#In_2_O_3_ nanofiber composite sensor were estimated at room temperature, and the response value of the composite sensor with an exposure of 1 ppm NH_3_ was 18.2, which was about 5.74 times larger than that of the pure PANI sensor. This composite sensor was demonstrated to be highly sensitive to the detection of NH_3_ ranging from the concentration between 0.6 ppm and 2.0 ppm, which is critical for kidney or hepatic disease detection from the human breath. The PANI/hollow C#In_2_O_3_ nanofiber composite sensor also displayed superior repeatability and selectivity at room temperature with exposures of 1.0 and 2.0 ppm NH_3_. Owing to the outstanding selectivity and repeatability of the detection of NH_3_ at 1.0 and 2.0 ppm confirmed in this investigation, the PANI/hollow C#In_2_O_3_ nanofiber composite sensor will be considered as a favorable gas-sensing material for kidney or hepatic disease detection from human breath.

## Figures and Tables

**Table 1:** A comparison of NH_3_-sensing properties of the PANI/hollow C#In_2_O_3_ nanofiber sensor established here, and the other sensors reported previously.

Materials	Gas	Conc.(ppm)	Temp. (°C)	Response	Ref.
PANI/In_2_O_3_	NH_3_	100	RT	3.2	[21]
PANI/ZnO	NH_3_	100	RT	2.5	[22]
PANI/TiO_2_-SiO_2_	NH_3_	50	RT	10	[23]
PANI/MoS_2_/SnO_2_	NH_3_	50	RT	7.5	[20]
PANI/Graphene/SnO_2_	NH_3_	10	RT	2.8	[24]
PANI/WO_3_	NH_3_	10	RT	7.14	[14]
PANI/PMMA	NH_3_	1	RT	1.4	[25]
PANI/PEO	NH_3_	1	RT	5	[26]
PANI/GNR/In_2_O_3_ nanoparticle	NH_3_	1	RT	10.3	[15]
PANI/N-GQD/hollow In_2_O_3_ nanofiber	NH_3_	1	RT	15.6	[16]
PANI/hollow C#In_2_O_3_ nanofiber	NH_3_	1	RT	18.2	This work
